# Gender Inequality in the Couple Relationship and Leisure-Based Physical Exercise

**DOI:** 10.1371/journal.pone.0133348

**Published:** 2015-07-21

**Authors:** Ellen Annandale, Anne Hammarström

**Affiliations:** 1 Department of Sociology, University of York, York, United Kingdom; 2 Department of Public Health and Clinical Medicine, Unit of Social Medicine, Umeå University, Umeå, Sweden; University of Rome, ITALY

## Abstract

**Aims:**

To analyse whether gender inequality in the couple relationship was related to leisure-based physical activity, after controlling for earlier physical activity and confounders.

**Methods:**

Data drawn from the Northern Swedish Cohort of all pupils in their final year of compulsory schooling in a town in the North of Sweden. The sample consisted of 772 respondents (n = 381 men, n = 391 women) in the 26-year follow-up (in 2007, aged 42) who were either married or cohabiting. Ordinal regression, for men and women separately, was used to assess the association between gender inequality (measured as self-perceived equality in the couple relationship using dummy variables) and a measure of exercise frequency, controlling for prior exercise frequency, socioeconomic status, the presence of children in the home, and longer than usual hours in paid work.

**Results:**

The perception of greater gender equality in the couple relationship was associated with higher levels of physical activity for both men and women. This remained significant when the other variables were controlled for. Amongst men the confidence intervals were high.

**Conclusions:**

The results point to the potential of perceived gender equality in the couple relationship to counteract the general time poverty and household burden that often arises from the combination of paid work and responsibility for children and the home, especially for women. The high confidence intervals among men indicate the need for more research within the field with larger samples.

## Introduction

Increasing physical activity (PA) in progressively sedentary societies has been a target of public health promotion policies and government health strategies across the global north. Although not without exception, existing research tends to show that men participate more than women do in most kinds of leisure-related PA, a reverse of many other health behaviours, such as smoking, alcohol consumption, and dietary choices where women’s lifestyles tend to be judged healthier than men’s [1,2]. For example, data from EU member states show that men are more likely to engage in sport and exercise than women, (especially when it is ‘vigorous’) as well as in ‘other’ PA (such as cycling, dancing). These gender differences are age-related, with higher male participation most marked in the 15–24 age-group and generally declining with age. There are also country-specific patterns. Thus those in the Nordic countries are the most likely of all member states to engage in sport and exercise on a regular basis [3]. In Sweden, the country considered here, leisure-time PA has increased more amongst women than amongst men of the same age (based on data for ages 40, 50 and 60) since the start of the 1990s [4].

There has been a growth of interest in the relation between gender, family, and leisure or free time (see e.g. [5]). Free time in particular has emerged as a key arena of ‘time inequality’ over the last 50 years or so as gender differences in time spent in paid work and in unpaid work, including household labour, have reduced (but not eroded) [6,7]. Even so research directly addressing leisure-based PA remains limited. Roos and colleagues [8] observed that men and women in Helsinki who reported ‘strong’ work-family conflicts were less likely to follow recommended amounts of PA. Family-work conflicts also showed a significant association, but only for women. Based on data for married couples in the US, Nomaguchi and Bianchi [9] found that when work and family roles were controlled for, women spent less time in PA than men. However, the negative effect of marriage on PA was larger for men than it was for women. The explanation provided by the authors is that marriage represents more of a change for men both in terms of their time use and sense of obligations. Specifically, the authors propose that the new importance of being a good father, husband and provider curtails men’s activity levels. Strong associations have also been found between frequency of PA and having dependent children (especially when they are aged five or below), especially for women [9,10,11]. This association is so marked that parenthood has been dubbed the ‘birth of inactivity’ [12].

Although research has drawn attention to ‘behavioural diffusion’ and the health practices of spouses and partners [2, 13], relatively little consideration has been given to the possible associations between quality of marriage and couple dynamics, and even less to perceived g*ender equality*, and health-related behaviours such as PA for those in couple relationships. One of the few analyses of the perceived equality in the couple relationship found psychological distress to be negatively influenced by gender inequality for both men and women [14]. But most research on gender equality has been directed at the macro-level looking at connections between health status and state or municipal-level gender equality as measured by factors such as income, political participation, and decision-making (see e.g. [15, 16]). It has been argued that for a more comprehensive account of gender equality and health we also need to pay attention to other levels, including that of the household, and to social norms and sanctions [17]. From this vantage, Sweden is of particular interest since its official gender equality policy states that women and men should have equal power to shape society and their own lives. Moreover, it emphasises that ‘gender equality is created where resources are distributed and decisions are made” (page 3) [18]. If cultural beliefs are an important component of the gender system, then social relational contexts, such as the couple relationship, are likely to be particularly salient since they are a key arena in which ‘gender equality’ is constituted and where beliefs or social rules play out [19]. Taking a lead from West and Zimmerman [20], in their research on couples and money in Sweden, Spain and the US, Halleröd and colleagues [21] not only found that ‘doing couple’ is informed by gender in a complex (and seemingly unconscious way), but that behaviour can be influenced by *how* ‘couple’ is ‘done’.

In sum, conceived as a gender relational context, perceived gender inequality in the couple relationship is likely to be an important resource or frame for action in relation to PA. It is reasonable to suppose that work overload is implicated in relationships perceived as *gender unequal*. Since women are more likely to experience overload [6, 22] it seems possible that their leisure activity will be impacted more than men’s. Contrariwise, it is likely that relationships perceived as more *gender-equal* will foster a sense of responsibility for the partner, as well as a greater willingness to cooperate and to negotiate how each other’s time is spent. Hence more gender-equal relationships may encompass a greater feeling of choice or latitude to engage in leisure-based PA for the individual (and engender more support for similar engagement by the partner in the relationship). Although it is also possible that feeling more gender-equal may prompt an individual to curtail their own leisure-based PA to give latitude to the partner in the relationship, we hypothesised that it is more likely that gender equality in the couple relationship is associated with higher levels of leisure-based PA for both women and men. However, given their generally higher time burden when work outside and inside the home is pooled [22], it is possible that women will benefit most from the practical latitude for PA that a more gender-equal relationship may confer.

The aim of this paper was to analyse whether gender equality in the couple relationship was related to leisure-based physical activity, after controlling for earlier physical activity and confounders.

## Methods

### Population

The population consists of all pupils in the last year of compulsory school in a middle-sized industrial town in Northern Sweden in 1981 (n = 1083). The participants have been followed with regular follow-ups and with an exceptionally high response-rate up to the most recent data collection when they were aged 42 in 2007 (94.5% of those still alive in the original cohort, n = 1010). The cohort has been followed with extensive questionnaires at ages 16, 18, 21, 30 and 42. For this paper, data from ages 21 and 42 are used on those 772 respondents (n = 381 men, n = 391 women) living in a couple relationship (i.e. combining married/cohabiting, as is usual in the collection and presentation of data in Sweden).

### Measures

There is an extensive literature on the most appropriate way to capture PA levels. Self-reports of frequency of PA over various periods such as the last week, last month and, less often, the last year, are common in survey research (see e.g. [3]). There are limitations to this approach since it does not capture the type, duration or intensity of PA [10]. Self-reports may also be subject to recall error. However since leisure-based exercise typically is undertaken in a planned and organised manner we can assume that it is easier to recall accurately than those activities which are habitual and hence less consciously undertaken (for example, walking as part of a daily routine, such as when doing the shopping, rather than consciously walking ‘for exercise’) [23]. Hence leisure-based PA was measured by how often respondents reported having engaged in exercise or sport *in the last 12 months* (‘daily’, ‘several times per week’, ‘sometimes per week’, ‘several times per month’, ‘sometimes per month’, ‘seldom/never’).

The exposure variable of *perceived level of gender equality* in the couple relationship was measured by the question ‘How gender-equal do you consider your couple relationship to be?’ on an ordinal scale of ‘totally gender-equal’, ‘quite gender-equal’, ‘somewhat gender- equal’, ‘not especially gender-equal’ or ‘not gender-equal at all’. The variable was recoded into three dummy variables: (1) ‘totally gender-equal’ versus all others, (2) ‘totally gender-equal’ and ‘quite gender-equal’ versus all others, and (3) ‘totally gender-equal’, ‘quite gender-equal’, ‘somewhat gender-equal’ versus ‘not especially gender-equal’ and ‘not gender-equal at all’. The skewed distribution was the reason why the two last answer alternatives were not separated into a fourth dummy variable. It is important to make clear that this question does not capture the normative dimension of gender equality. It should not be assumed, for example, that those who reported less gender-equal relationships necessarily found this unfair or problematic (though they might well have done). Nor does it tell us whether the perceived inequality was in the direction of lesser or greater power for the respondent (male or female) in the relationship. Nevertheless, the variable is an innovative measure of self-perceived inequality and has been used in previous research [24, 25].

We also controlled for several factors which may confound the relationship between gender equality and leisure-based PA. These were: *prior exercise at age 21* (reported in 1986) using the same measure of frequency of exercise over the last 12 months already referred to; *socio-economic status at age 42*, using the official Swedish occupation-based measure (entrepreneurs and farmers, upper white collar, lower white collar, blue collar); *number of children living in the home* (none, 1 or more) at age 42; and long hours in paid work (engaging in paid work for more than 40 hours per week, or ‘*overtime*’).

#### Statistical analysis

For the statistical analyses, the proportional odds model was used. Ordinal regression was chosen because the dependent variable had an ordinal scale. The analyses were first performed separately for the main independent variable, after which all confounders were added in the multivariate analysis. The correlation between the confounders was < 0.3. A significance level of <0.05 was chosen. There was a significant interaction between perceived gender inequality and gender and, therefore, the results are presented separately for men and women.

The estimates (log odds) were transformed into odds ratios. The proportional odds assumption was tested using the test of parallel lines. We concluded that the lines were parallel and thus the conditions were fulfilled. IBM-SPSS was used for all statistical analyses.

#### Ethical statement

The Regional Ethical Review Board in Umeå, Sweden, has approved the study. Written consent has not been requested from the Board because, according to Swedish law (Swedish Ethical Review Act 2003; 460, §17), there is no requirement of written consent in a questionnaire study. The respondent is regarded as giving written consent when answering the questionnaire. Participants were/are able to opt out at any time simply by not completing any wave(s) of the survey.

## Results

The data in Table 1 show that although more men described their couple relationship as ‘totally gender-equal’ and, conversely, more women reported it as ‘somewhat’, ‘not especially’ and ‘not at all’ gender-equal (with similar reports of ‘quite’ gender-equal), this was not significant. In common with much existing research, men reported a higher frequency of prior PA (over the last 12 months) when they were aged 21. However, contrary to expectations, it was women who had engaged in more frequent PA in the last 12 months at age 42. The data on socioeconomic position revealed significantly more men in the highest category of entrepreneurs and farmers and far more women in lower white collar occupations, such as office work. There were no significant differences in whether there were children in the home or not. Significantly more men than women worked ‘overtime’.

**Table 1:** Distribution of dependent and independent variables among men and women, per cent or mean.

	Men %	Women %	P value*
**Self-perceived inequality in couple relationship**			0.066
Totally gender equal	42.7	36.7	
Quite gender equal	40.3	40.1	
Somewhat gender equal	13.6	15.8	
Not especially gender equal	3.1	6.2	
Not at all gender equal	0.3	1.3	
**Physical activity in last 12 months (at age 42)**			<0.001
Daily	6.5	11.3	
Several times a week	25.8	31.1	
Sometimes a week	23.8	22.9	
Several times per month	7.8	6.7	
Sometimes per month	13.8	13.1	
Seldom/never	22.2	14.9	
**Physical activity in last 12 months (at age 21)**			<0.001
Daily	9.4	8.0	
Several times a week	25.2	17.4	
Sometimes a week	22.6	21.8	
Several times per month	10.0	7.3	
Sometimes per month	10.2	24.4	
Seldom/never	22.6	21.2	
**Socioeconomic position (at age 42)**			<0.001
Entrepreneurs and farmers	13.8	4.1	
Upper white collar	43.3	45.3	
Lower white collar	8.4	18.3	
Blue collar	34.5	32.4	
**Children in home (at age 42)**	86.9	90.2	0.17
**Working overtime (at age 42)**	34.4	13.3	<0.001

*Differences between men and women tested for significance by chi-square for categorical variables and t test for continuous variables

Tables 2 and 3 present the ordinal regression analyses of perceived gender equality on PA in the ‘last 12 months’, separately for women and men. There were no significant results for the first two dummy variables. The results for the third dummy variable (comparing ‘totally gender-equal’, ‘quite gender-equal’ and ‘somewhat gender-equal’, with ‘not especially gender-equal’ and ‘not gender-equal at all’) are shown in the Tables. The data show that moving from a lower to higher degree of perceived gender equality increased the odds for PA among both women and men in all models. Among men the confidence intervals were wide, indicating a low degree of statistical power. Overall, the pseudo r2 were quite low in all models.

**Table 2:** Ordinal regression analysis for physical activity for women in relation to increasing degree of gender equality in the couple relationship after controlling for confounders (odds ratio OR and 95% confidence intervals CI).

	Model 1		Model 2		Model 3		Model 4	
	OR	CI	OR	CI	OR	CI	OR	CI
Gender equality	2.091	1.067–4.098	2.117	1.048–4.278	2.182	1.078–4.417	2.136	1.055–4.327
Pseudo r2	0.13		0.11		0.13		0.13	

Model 2 controls for prior exercise; Model 3 controls for model 2+ SES; Model 4 controls for model 3+children at home+overtime

**Table 3:** Ordinal regression analysis for physical activity for men in relation to increasing degree of gender equality in the couple relationship after controlling for confounders (odds ratio OR and 95% confidence intervals CI).

	Model 1			Model 2		Model 3		Model 4	
	OR	CI		OR	CI	OR	CI	OR	CI
Gender equality	3.44	1.236–9.597	11.16	4.007	1.415–11.346	3.936	1.388–11.160	3.78	1.335–10.697
Pseudo r2	0.12			0.12		0.12		0.15	

Model 2 controls for prior exercise; Model 3 controls for model 2+ SES; Model 4 controls for model 3+children at home+overtime

## Discussion and Conclusions

We found that gender equality in the couple relationship was related to higher levels of PA amongst both men and women. The high confidence intervals among men indicate the need for more research within the field with a larger sample. Significant results were only found for the dummy variable comparing answer alternatives for the two most unequal couple relations (i.e. combined ‘not especially gender-equal’ and ‘not gender-equal at all’) with relations that were defined as ‘totally’, ‘quite’ and ‘somewhat’ gender-equal. These findings indicate that is it important to distinguish the near eight per cent of women and just over three per cent of men reporting a less gender-equal couple relationship from the large majority who reported a more equal relationship.

Since, as already noted, Sweden has a national-level gender equality policy defined as women and men having “equal power to shape society and their own lives” (page 2) [18], we might expect a much greater degree of diffusion of gender justice to the micro level of the cohabiting couple relationship than in many other countries. The results clearly show that even though there were no statistically significant differences in men’s and women’s perceptions of gender equality in their couple relationship, perceived gender equality was positively associated with PA for both. Since prior PA participation (at age 21) was strongly associated with present PA, the fact that it does not take away the significance of gender equality at age 42 suggests that it has an important independent association with PA.

Our hypothesis that perceived equality in the couple relationship would be associated with higher levels of PA for both men and women was therefore supported by the analysis. The reasons for this cannot be directly ascertained from the data, but, as noted earlier, we suggest that couple relationships that are perceived to be more gender-equal are likely to be more cooperative and to foster greater choice and latitude for the individual through greater felt responsibility for the well-being of one partner by the other. Although it is not clear cut, prior research has suggested that marriage/a couple relationship may benefit the health and health-related behaviours of men more than it does women [2]. This was not borne out in terms of PA here since women engaged in more PA than men. As already noted, leisure time PA in Sweden has increased more among women than men (aged 40, 50, 60) since the early 1990s. By 2007 (the most relevant year for the present results) this had resulted in at least gender parity, or even higher rates, amongst women for some ages. Swedish data for ages 16–84 show that being ‘physically active’ (defined as regular physical activity at least twice a week) increased from around 25% of 16–84 year olds in 1980 (where men were somewhat more active than women) to around 60% in 2009 (when women were more active than men) [4]. The results of the present analysis of a northern cohort may tentatively point to the possibility that perceived gender equality can counteract the general time poverty and household burden that often arises for women from paid work alongside responsibilities for children and the household. It should be noted that we are unable to determine if all of the couple relationships were heterosexual or if some were same sex. Previous research suggests that gay and lesbian civil partnerships and marriages tend to be more egalitarian than heterosexual relationships in terms of the division of domestic labour, and paid work time [26], as well as in ‘relationship maintenance behaviours’ such as seeing each other’s point of view [27]. While this is not the same conceptualisation of gender equality in the couple relationship as used here, if same sex couple relationships are experienced as more gender equal they may especially foster higher levels of engagement in PA. More research is needed to explore this possibility.

More generally, the findings lend support to the recent emphasis placed by social scientists on the importance of taking a gender relations approach to research on marriage, couple relationships and the family [28, 29]. Research has shown consistently that the health-related behaviours of individuals and ‘health lifestyles’ are shaped by the contexts within which they are formulated and enacted [30]. As Bird and Rieker explain, “many men and women cannot effectively make their own health a priority because they are distracted or overwhelmed by obligations that compete for their resources, energy, and attention” (pages 173–4) [31]. Gender orders–socially patterned relationships between men and women–are part of “a dense and active social tissue of institutions and sites” (page 1677) [32]. They operate simultaneously at institutional and society-wide levels and, crucially, at the level of everyday social practices, which include family and couple relationships [19, 32]. As might be expected in such contexts we have found that gender relations at the interpersonal level of the ‘couple relationship’ appear to have a particular influence on the extent to which both women and men engage in leisure-based PA which can be considered a form of health-promoting behaviour.

### Strengths and limitations

The main strength of this study is its capacity to bring the concept of gender equality into the study of leisure-based PA and to do so by the use of an innovative measure of perceived equality in the couple relationship. The use of a follow-up data set with an exceptionally high response rate (and hence minimal non-response bias) at each contact point (age 21, age 42) enables us to control for prior PA. However, although we employ the kind of measure of exercise frequency routinely used in the wider literature, we lack data on the type, duration, intensity or quality of PA, which arguably may vary by gender. For leisure generally it has been suggested that women’s free time and leisure activity may be less ‘pure’ than men’s. In other words, it is likely to be more ‘contaminated’ by other simultaneous activities, such as those involving others, including children [33, 34], though this seems more likely to be related to leisure activities such as watching television or reading than to most types of PA. It should also be noted that women and men have been found to participate in different kinds of exercise for a wide range of reasons [1, 9, 35]. Thus we should not assume that those in the present sample consciously engage in PA for health reasons, though PA may have benefits for health and wellbeing nonetheless.

Confounders are always important to consider. Our exposure was gender inequality in the couple relationship. At age 42 most people in relationships in Sweden live together [18] and, therefore, our population was limited to those who were married/cohabitating. Prior research has pointed strongly to the impact of children in the home upon adult PA [9, 10, 11, 12]. Our data set allowed us to include the presence or absence of children in the home in the analysis, but not their number and ages. Certain handicapping conditions, such as being a wheelchair user, could be a confounder influencing both the domestic work and childcare that can be performed and the possibility of engaging in PA. However, in this relatively young cohort, there was only one person who, as a result of tetraplegia, used a wheelchair. We use respondents’ own socioeconomic position as a possible confounder. Earlier research has shown that the division of domestic work (which we were unable to address in the analysis) tends to be more equal when the couple’s socioeconomic position is equal or when women have a higher socioeconomic position than the partner [36]. Moreover, there is evidence that as women’s earnings increase to the point where both spouses contribute equally women’s housework decreases. For example Bittman and colleagues found that where women contribute 51 per cent or more, a form of compensation seems to occur. A more traditional division of labour transpires and women’s burden is higher [37]. Thus, relative socioeconomic status and earnings may be important to analyse in future research on possible mechanisms between gender inequality and PA. Finally, it should be said that our data are silent on *how* the micro-dynamics of the couple relationship may influence PA. Extrapolating from Halleröd and colleagues’ [21] study of the dynamics of money, it is reasonable to assume the actions that flow from the couple relationship, such as engaging in PA, are not necessarily the result of rational and explicit power relations, but rather may seem to ‘just happen’ when ‘doing couple’. Teasing out these dynamics is an important area for future study.

## References

[1] Payne S (2006) The Health of Men and Women. Cambridge: Polity p. 229

[2] Carr D, Springer K (2010) Advances in families and health research in the 21^st^ century. Journal of Marriage and Fam 72:743–61.

[3] European Commission (2014) Special Eurobarometer 414 Sport and Physical Activity. P. 135 Available: http://ec.europa.eu/public_opinion/archives/ebs/ebs_412_en.pdf.

[4] **F**olkhälsorapport 2014 [Public Health Report 2014] [Internet] (2014). Stockholm: Socialstyrelsen [The National Board of Health and Welfare] Available: https://www.folkhalsomyndigheten.se/pagefiles/17825/Folkhalsan-i-Sverige-arsrapport-2014.pdf. Accessed May 2015.

[5] Bittman M, Wajcman J (2000) The rush hour: the character of leisure time and gender equity. Soc Forces 79:165–189.

[6] Bianchi R, Milkie M (2010) Work and family research in the first decade of the 21^st^ century. Journal of Marriage and Fam 72: 705–25.

[7] Sayer LC (2005) Time and inequality: trends in women’s and men’s paid work, unpaid work, and free time. Soc Forces 84:285–303.

[8] Roos E, Sarlio-Lähteenkorova LT, Lahelma E (2007) Associations of work-family conflicts with food habits and physical activity. Public Health Nutr 10: 222–229 17288618 10.1017/S1368980007248487

[9] Nomaguchi K, Bianchi S (2004) Exercise time: gender differences in the effects of marriage, parenthood and employment. Journal Marriage Fam 66: 413–430.

[10] Brown H, Roberts J (2011) Exercising choice: the economic determinants of physical exercise activity behaviour of an employed population. Soc Sci Med 73: 383–390. 10.1016/j.socscimed.2011.06.001 21757272 PMC3162476

[11] Such E (2008) Leisure and fatherhood in dual-earner families. Leisure Studies 25: 185–199.

[12] Bellows-Riecken K, Rhodes RA (2008) Birth of inactivity? A review of physical activity and parenthood. Prev Med 46: 99–110. 17919713 10.1016/j.ypmed.2007.08.003

[13] Lewis AM, McBride CM, Pollak KI, Puleo E, Butterfield RM, Emmons KM (2006) Understanding health behaviour change among couples: an independence and communal coping approach. Soc Sci Med 62: 1369–1380. 16146666 10.1016/j.socscimed.2005.08.006

[14] Harryson L, Novo M, Hammarström A (2012) Is gender inequality in the domestic sphere associated with psychological distress among men and women? Results from the Northern Swedish Cohort. J Epidemiol Community Health 66: 271–276. 10.1136/jech.2010.109231 20940171

[15] Backhans M, Lundberg M, Månsdotter A(2007). Does increased gender equality lead to a convergence of health outcomes for men and women? A study of Swedish municipalities. Soc Sci Med 64: 1892–1903. 17339070 10.1016/j.socscimed.2007.01.016

[16] Van de Velde S, Huijts T, Bambra C, Bracke P (2013) Macro-level equality and depression in men and women in Europe. Sociol Health Ill 35:682–698. 10.1111/j.1467-9566.2012.01521.x 23145770

[17] Moss N (2009) Gender equity and socioeconomic inequality: a framework for the patterning of women’s health. Soc Sci Med 54: 649–61. 10.1016/s0277-9536(01)00115-0 11999484

[18] Statistics Sweden (2014) Men and Women in Sweden 2014. Avaialble: http://www.scb.se/en_/Finding-statistics/Publishing-calendar/Show-detailed-information/?publobjid=22161+. Accessed June 2015.

[19] Ridgeway C, Correll S (2004) Unpacking the gender system: a theoretical perspective on gender beliefs and social relations. Gender Soc 18: 510–31.

[20] West C, Zimmerman D (1987) Doing Gender. Gender Soc 1:125–151

[21] Halleröd B, Díaz C, Stocks J (2007) Doing gender while doing couple: concluding remarks In: Stocks J, Díaz C and Halleröd B (editors) Modern Couples Sharing Money, Sharing Life. Basingstoke: Palgrave p. 196

[22] Davis S, Greenstein T (2013) Why study housework? Cleaning as a window into power in couples. Journal of Family Theory and Review 5:63–71.

[23] National Centre for Health Research (2009) *Health* Survey for England Vol 1 Physical Activity and Fitness. Leeds: National Centre for Health Research.

[24] Nordenmark M, Nyman C (2003) Fair or unfair? Perceived fairness of household division of labour and gender equality among women and men: The Swedish case. European Journal of Women’s Studies 10: 181–209.

[25] Harryson L (2013) ‘An equal share, that’s my medicine’ Work, gender relations and mental illness in a Swedish context. Umeå, Umeå University.

[26] Biblarz TJ, Savci E (2010) Lesbian, Gay, Bisexual, and Transgender Families. Journal of Marriage and Fam 72:480–497

[27] Solomon SE, Rothblum ED, Balsam KF (2005) Money, housework, sex, and conflict: same-sex couples in civil unions, those not in civil unions, and heterosexual married siblings. Sex Roles 52: 561–575

[28] Grzywacz J, Marks N (2000) Family, work, work-family spillover, and problem drinking during midlife. Journal Marriage Fam 62:336–348.

[29] Umberson D, Williams K, Powers D, Liu H, Needham B (2006). ‘You make me sick’: marital quality and health over the lifecourse. Journal Health Soc Behav 47:1–16. 16583772 10.1177/002214650604700101 PMC3149975

[30] Cockerham W (2005) Health lifestyle theory and the convergence of agency and structure. J Health Soc Behav 46: 51–67. 15869120 10.1177/002214650504600105

[31] Bird C, Rieker P (2008) Gender and Health. Cambridge: Cambridge University Press p. 256.

[32] Connell R (2012) Gender, health and theory: conceptualizing the issue, in local and world perspective. Soc Sci Med 74: 1675–1683. 10.1016/j.socscimed.2011.06.006 21764489

[33] Ferree MM (2010) Filling the glass: gender perspectives on families. Journal Marriage and Fam 72:420–439.

[34] Mattingly MJ, Bianchi SM (2003) Gender differences in quantity and quality of free time: the U.S. experience. Soc Forces 81:999–1030.

[35] Markula P, Kennedy E (2011) Beyond binaries: contemporary approaches to women and exercise’ In: Kennedy E. and Markula P. (editors) Women and Exercise. The Body, Health and Consumerism. London: Routledge p.320.

[36] Evertsson M, Nermo M (2007) Changing resources and the division of housework: a longitudinal study of Swedish couples. Euro Sociol Rev 23: 455–70.

[37] Bittman M, England P, Sayer L, Folbre N, Matheson G (2003) When does gender trump money? Bargaining and time in household work. Am J Sociology 109: 186–214.

